# Weighted Vest Combined With Vibrotactile Stimulations Decrease the Sympathetic Activity: A Repeated Measures Study

**DOI:** 10.1002/hsr2.70194

**Published:** 2024-11-13

**Authors:** Mohamad Izzur Maula, Muhammad Imam Ammarullah, Chandra Maulana Nugwita, Muhammad Sultan Faisal, Ilham Yustar Afif, Farhan Ali Husaini, M. Danny Pratama Lamura, Jamari Jamari, Tri Indah Winarni

**Affiliations:** ^1^ Undip Biomechanics Engineering & Research Centre (UBM‐ERC) Universitas Diponegoro Semarang Central Java Indonesia; ^2^ Department of Mechanical Engineering, Faculty of Engineering Universitas Diponegoro Semarang Indonesia; ^3^ Department of Manufacturing Engineering Technology Akademi Inovasi Indonesia Salatiga Central Java Indonesia; ^4^ Department of Mechanical Engineering, Faculty of Engineering Universitas Muhammadiyah Semarang Semarang Central Java Indonesia; ^5^ Department of Anatomy, Faculty of Medicine Universitas Diponegoro Semarang Central Java Indonesia; ^6^ Center for Biomedical Research (CEBIOR), Faculty of Medicine Universitas Diponegoro Semarang Central Java Indonesia

**Keywords:** anxiety, Comfort Rating Scale, skin conductance, pulse rate, deep pressure therapy, stress, vibrotactile

## Abstract

**Background and Aims:**

Mental and neurological disorders are a growing global concern, further intensified by the COVID‐19 pandemic. Stress management techniques, such as deep pressure therapy, have gained attention, with weighted vests commonly used for anxiety relief. However, there is limited scientific evidence supporting their efficacy. This study aimed to rigorously assess the effectiveness of a weighted vest incorporating vibrotactile stimulation in reducing anxiety by measuring physiological indicators—pulse rate and skin conductance (SC)—as well as subjective comfort ratings.

**Methods:**

A total of 30 final‐semester college students participated in the study. Participants wore a vibrotactile‐weighted vest, and both pulse rate and skin conductance (SC) were measured to gauge anxiety levels. Additionally, participants rated their comfort using a Comfort Rating Scale (CRS). Changes in pulse rate and SC were statistically analyzed, and effect sizes (Cohen's *d*) were calculated to assess the magnitude of the intervention's impact.

**Results:**

The weighted vest with vibrotactile stimulation resulted in a significant reduction in both pulse rate (dpulse = 0.23–0.62) and SC (dsc = 0.32–0.66), indicating a small to medium effect size in anxiety reduction. Subjective evaluations of the vest using the CRS revealed low scores on discomfort‐related items, with participants rating unfavorable statements between 1.5/10 and 4.6/10, suggesting overall comfort during use.

**Conclusion:**

This study provides compelling evidence that vibrotactile‐weighted vests effectively reduce anxiety, as indicated by both physiological measures and subjective comfort ratings. The findings support the potential of this intervention as a formal therapeutic tool for stress and anxiety reduction. Further research may explore long‐term effects and broader applications in clinical settings.

## 1 Introduction

Nearly a billion individuals worldwide suffer from mental, neurological, and drug use disorders. Moreover, the world is being hit by the COVID‐19 pandemic outbreak which has severely impacted all sectors including mental health then led to a rise in anxiety, depression, and substance misuse [1]. There are at least 53.2 million new cases (prevalence 3.1/100) of major depressive disorder and 76.2 million new cases (prevalence 4.8/100) of anxiety disorders globally due to the COVID‐19 pandemic [2]. Mental health problems, such as stress, can also be caused by other variables. Adolescents in developing countries report that financial problems and social pressures are related to urban stress [3]. Another study also reported that the burden of education also affects stress, especially in college students [4, 5].

The World Health Organization has labeled stress a “worldwide epidemic” in recognition of the magnitude of its damaging effects [6]. One of the cornerstones of a happy life is health which includes two important dimensions, mental health and physical health [7]. Diseases are not only in the body but also include diseases that affect psychology and behavior. Even this mental illness has the potential to cause a more serious internal disease. If several things trigger the occurrence of mental health disorders, it will cause several other disorders such as stress, anxiety, and other mental health disorders. However, there are still very few resources available, particularly in low‐ and middle‐income countries, for the diagnosis, treatment, and support of people with mental health difficulties [1].

Stress comes in various emotional, mental, and physical forms. Something that causes stress is called a stressor. When a stressor is overwhelming and cannot be resolved, stress becomes chronic. Chronic stress is linked to macroscopic changes in certain brain areas, potentially causing cognitive, emotional, and behavioral dysfunctions, and may increase vulnerability to psychiatric disorders. Most studies also focused on the effects of stress on the neuroimmune system, so that stress and other psychological illnesses will have an impact on physical illness [8].

Treating anxiety, stress, and depressive disorder is the tricky part, but what can be done is to reduce stress and anxiety levels. One way to reduce stress levels is to create a proper stimulation of peripheral tissue such as skin and muscle [8]. Some of the proper stimulation that can give a calming effect to the body is deep pressure and vibration [9, 10]. Deep pressure is the application of certain pressure and for a certain time in‐depth to most of the outside of the body evenly, to provide a calming effect simultaneously, reducing uncontrolled physical activity due to anxiety [11]. A sense of touch or tactile stimulation is also provided by deep pressure, providing an enjoyable, effective, and widely used to relieve anxiety [12]. It has been reported that deep pressure, such as rolling over on a mat, has been used to calm children with autism and ADHD disorders, and the foam brace‐induced deep pressure touch arm reduced behavior self‐injury and self‐stimulation in children with autism disorder [13].

Autism Hug Machine Portable Seats (AHMPS) as new models of deep pressure tools were introduced by Afif et al. [14] and reported to have positive effects in stimulating calming. However, this chair‐shaped device greatly limits movement because it binds the user's chest and thighs. This limitation can be covered by other forms of deep pressure tools, one of the most widely used is the weighted vest. The simple and very portable shape of the vest makes it can be applied in many conditions. Nonetheless, Taylor et al. [11], many studies on the use of weighted vests still have not found a consensus on their efficacy. According to Seco et al. [15], passive pressure may be involved in this inconsistency and suggested active intervention may be applied to the vest.

Vibration therapy that applies active vibrations to the body is gaining traction as an alternative treatment. Vibration therapy has been reported to stimulate hormonal responses and improve quality of life to increase strength, muscle mass, and flexibility to improve balance, postural control, and blood flow, and to reduce chronic pain [15]. Uzsen et al. conducted a randomized controlled trial and found that using vibration and pressure interventions during intramuscular injections significantly reduced pain, anxiety, and fear in children in the emergency department, as reported by children, mothers, and nurses [16]. Additionally, according to Field's review of massage therapy research, localized vibration therapy applied to specific muscle groups has been found to decrease anxiety and improve mood in individuals with fibromyalgia [17]. Combining vibration with the deep pressure provided by the weighted vest is expected to increase the efficacy of the treatment in reducing stress and anxiety.

Based on scientific evidence, this study aims to (i) evaluate the efficacy of the weighted vest with vibrotactile stimulation in reducing stress objectively and (ii) conduct a subjective comfort test of the device. We hypothesize that (i) the weighted vest with vibrotactile stimulation will reduce sympathetic activities as physiological markers of stress and (ii) the vibrotactile weighted vest is a comfortable device. This study contributes to providing novelty to the study of the efficacy of deep pressure both subjectively and objectively, especially through the modification of a weighted vest combined with vibrotactile stimulation.

## 2 Materials and Methods

### 2.1 Study Design and Participants

A repeated measures experimental study was conducted to investigate the effects of a weighted vest with and without vibrotactile stimulation during the final semester with two steps, objective pretest–posttest evaluation of its effect in reducing physiological stress and subjective measurements to evaluate the comfort of the prototype. Participants underwent all conditions (several vibration patterns and without vibration as a control) on separate days in a counterbalanced order. The counterbalanced design, where the order of conditions was varied across participants, was implemented to mitigate potential order effects, such as practice or fatigue, that could confound the results. Additionally, administering the conditions on separate days ensured a sufficient washout period, minimizing the potential carryover effects of one condition influencing the subsequent condition. Participants who took this experiment had the same criteria as the previous study conducted by Foo et al. [18], which were limited to male adolescents, 18–27 years old, dress size M–L (Asian size), height 160–180 cm, and physically fit.

### 2.2 Weighted Vest With Vibrotactile

**Figure 1:**
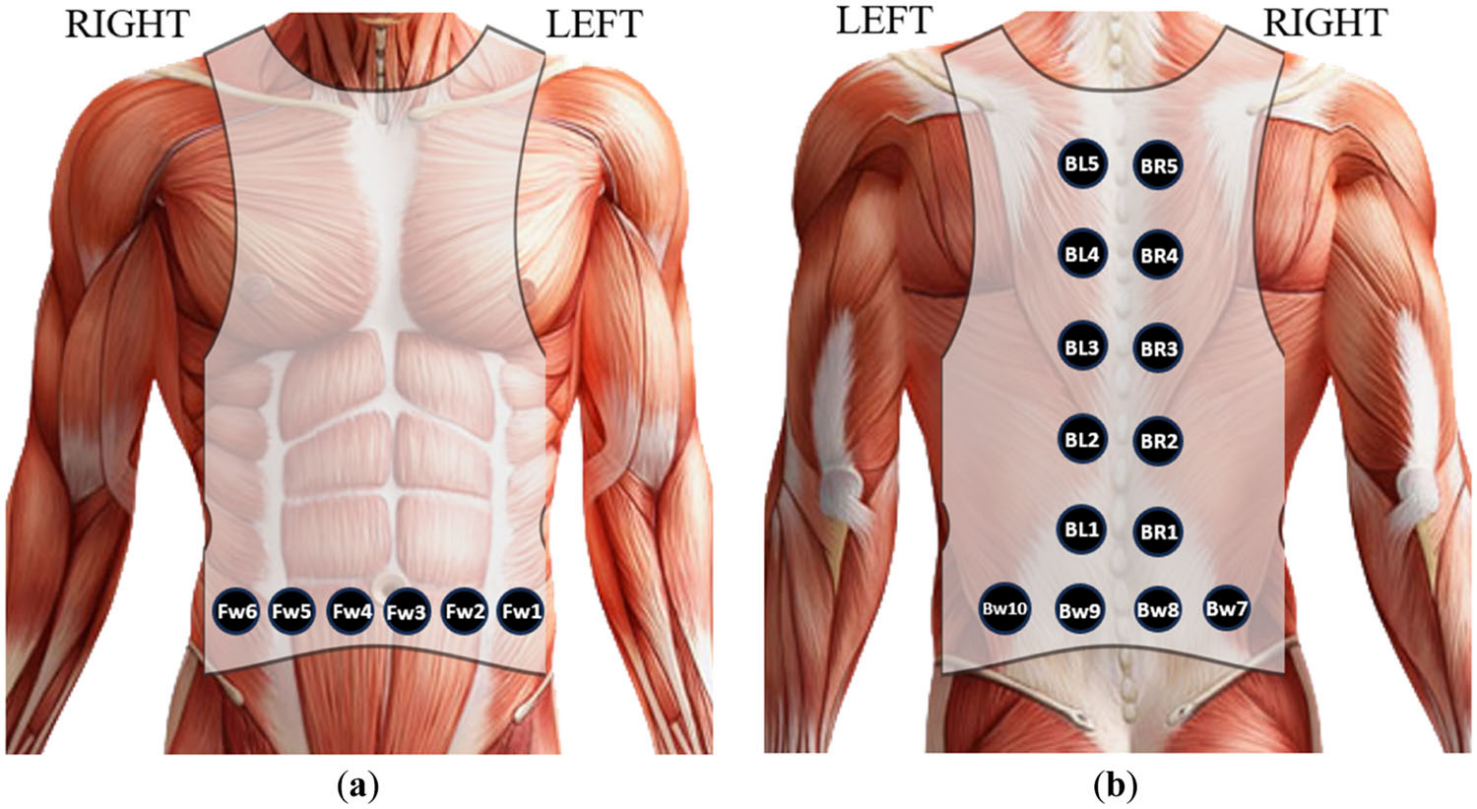
Vibrating actuators placements: (a) front and and (b) back.

In this study, a modified weighted vest equipped with vibrators was utilized. Vibrotactile stimulation is generated by 20 vibrators placed on the vest, following specific points as indicated in Figure 1.

These points were adopted and modified from a previous study conducted by Morrison et al. [19]. The vibrators are controlled wirelessly through a Bluetooth connection to a smartphone, providing four distinct vibration directions, as specified in Table 1.

**Table 1:** Vibration pattern and direction.

Pattern	Vibrating direction
Up	Bw8, Bw9 → BL1, BR1 → BL2, BR2 → BL3, BR3 → BL4, BR4 → BL5, BR5
Down	BL5, BR5 → BL4, BR4 → BL3, BR3 → BL2, BR2 → BL1, BR1 → Bw8, Bw9
WFTB	Fw3, Fw4 → Fw2, Fw5 → Fw1, Fw6 → Bw10, Bw7 → Bw9, Bw8
WLTR	Fw1 → Fw2, Bw10 → Fw3, Bw9 → Fw4, Bw8 → Fw5, Bw7 → Fw6

Abbreviations: BL = back left; BR = back right; Bw = back waist; Fw = front waist; WFTB = waist front to back; WLTR = waist left to right.

### 2.3 Measurement Tools

#### 2.3.1 Comfort Rating Scale (CRS)

CRS which consists of five statements was used as a subjective measurement. The five statements represent various dimensions of a person's comfort when wearing something on their body and rate them on a scale of 1 (strongly disagree) to 10 (strongly agree) adapted and modified from Knight and Baber [20]. The statement to be filled in as a comfort assessment is shown in Table 2.

**Table 2:** Comfort Rating Scale statements.

Parameters	Statements
Emotion	I am worried about how I look when I wear this device. I feel tense or on edge because I am wearing the device.
Harm	The device is causing me some harm. The device is painful to wear.
Perceived change	Wearing the device makes me feel physically different. I feel strange wearing the device.
Movement	The device affects the way I move. The device inhibits or restricts my movement.
Anxiety	I do not feel secure wearing the device.

In this rating scale, all five statements queried represent unfavorable responses. Hence, a higher rating on the CRS indicates the participant's discomfort while using the device, and vice versa.

#### 2.3.2 Peripheral Pulse Sensor

An Elitech FOX‐1 pulse oximetry (Elitech Corp., Indonesia) was used in this study to measure pulse/heart rate. In this sensor, light rays are emitted or reflected into the bloodstream to detect heart rate variability using photoplethysmography (PPG). The change in light energy, therefore, is interpreted as a cardiac cycle in relation to the systole and diastole of the heart [21, 22].

Pulse rate was selected as a physiological measurement tool due to its user‐friendly nature and rapid results. Despite the distinction between peripheral pulse and heart rate, studies have shown a robust correlation between the two. This choice was made based on the convenience and efficiency of pulse oximetry, even though it measures peripheral pulse, as it offers quick and reliable results [23].

#### 2.3.3 Galvanic Skin Response (GSR) Sensor

A Shimmer GSR sensor (Shimmer Ltd., Ireland) was used in this study to measure skin conductance. Skin conductance, a physiological outcome of electrodermal activity (EDA), is a commonly measured indicator used to assess the activation of the autonomic nervous system (ANS). This measurement reflects the skin's electrical properties influenced by sweat gland activity and has been widely employed to gauge ANS activity, specifically the sympathetic nerves associated with high arousal [24, 25]. Recognized as a highly sensitive and valid biomarker, skin conductance serves as a valuable tool for the noninvasive evaluation of emotional stress and arousal levels. A decrease in skin conductance indicates a reduction in stress and arousal levels [18, 26].

### 2.4 Procedures

On the first day, participants were briefed about the study and requested to sign a consent form before the commencement of the research. Each participant received five different treatments: Up, Down, WFTB, WLTR, and without vibration (control group). To avoid confounding variables arising from different treatments, each treatment was administered on separate days. Each treatment session lasted for 5 min, during which pulse rate and skin conductance were measured both before (pretest) and after (posttest) the intervention. On the fifth day following the completion of all sessions, participants will be required to fill out the CRS. The entirety of these studies was conducted at the Undip Biomechanics & Engineering Research Center (UBM‐ERC), Universitas Diponegoro.

### 2.5 Statistical Analysis

The Shapiro–Wilk test was employed to assess the normality of the distribution of the physiological data (pulse rate and skin conductance) within each condition, as the sample size was small (*N* < 50) [27]. This was necessary to determine the appropriateness of using parametric statistical tests, such as the paired *t*‐test, which assume normality. A paired *t*‐test was applied to estimate the significant changes in physiological data in the pretest–posttest. This test directly addressed our hypothesis that vibrotactile stimulation would lead to a significant decrease in physiological arousal compared to before intervention. The level of significance was set at *α* = 0.05 for all analyses, and all tests were two‐sided. Significant decreases in pulse rate and skin conductance demonstrated the device's calming effect.

Cohen's *d* was calculated to quantify the effect size of vibrotactile‐weighted vest uses. This test was selected because of its suitability for estimating the effect of paired pretest–posttest treatment. The effect size (*d*) was defined in Equation (1), as follows:

(1) $$d=\frac{\left({\mathrm{Mean}}_{\mathrm{post}}-{\mathrm{Mean}}_{\mathrm{pre}}\right)}{S}J,$$

where *J* is the correction factor for a small sample size (*N*) expressed in Equation (2) and *S* is the pooled standard deviation shown in Equation (3).

(2) $$J=\left(\frac{N-3}{N-2.25}\right)\left(\sqrt{\frac{N-2}{N}}\right),$$

(3) $$S=\sqrt{\left[\frac{\left({\mathrm{SD}}_{\mathrm{pre}}^{2}+{\mathrm{SD}}_{\mathrm{post}}^{2}\right)}{2}\right]}.$$

In Cohen's *d* interpretation, in general, 0.2 ≤ *d* < 0.5 is considered small, 0.5 ≤ *d* < 0.8 is medium, and *d* ≥ 0.8 is large [28]. All statistical analyses were performed using IBM SPSS Statistics version 27 (IBM Corp., Armonk, NY).

## 3 Results

This study involved 30 participants who met the specified criteria and willingly consented to participate in a comprehensive series of research sessions. A carefully selected sample of participants was chosen based on specific criteria to ensure the study's reliability is maintained as well as the quality of the data collected during the study.

### 3.1 Physiological Changes

#### 3.1.1 Pulse Rate

**Figure 2:**
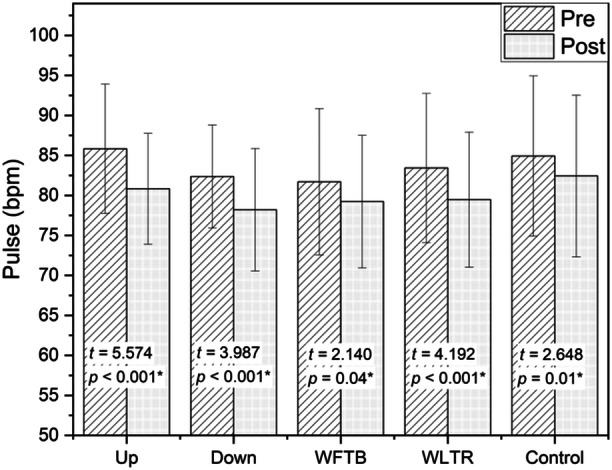
Pretest–posttest changes in pulse rate.

A significant decrease in pulse rate (*p* < 0.05) occurred in all types of treatments, as indicated in Figure 2. The up pattern exhibited the highest mean difference in pulse rate reduction, followed by Down, WLTR, and WFTB, respectively. Detailed information on the changes in pulse rate among the 30 participants and the corresponding effect sizes for each treatment can be found in Table 3.

**Table 3:** Effect size of pretest–posttest changes in pulse rate.

	*N*	Pre mean (SD)	Post mean (SD)	Mean difference (95% CI)	*t*	*p*	*d*	Effect size
*Pulse*								
Up	30	85.83 (8.09)	80.83 (6.94)	5.00 (3.17, 6.83)	5.547	< 0.001*	0.62	Medium
Down	30	82.37 (6.44)	78.20 (7.65)	4.17 (2.03, 6.30)	3.987	< 0.001*	0.55	Medium
WFTB	30	81.70 (9.15)	79.23 (8.29)	2.47 (0.11, 4.82)	2.140	0.041*	0.27	Small
WLTR	30	83.43 (9.33)	79.47 (8.44)	3.97 (2.03, 5.90)	4.192	< 0.001*	0.42	Small
Control	30	84.93 (10.01)	82.43 (10.10)	2.50 (0.57, 4.43)	2.648	0.013*	0.23	Small

* *p* ≤ 0.05 (significant).

#### 3.1.2 Skin Conductance

In this variable, 11 out of 30 skin conductance measurement data were not captured accurately due to reasons unknown. There was a possibility that human error occurred during recording and/or data storage, resulting in only 19 participant data being analyzed.

**Figure 3:**
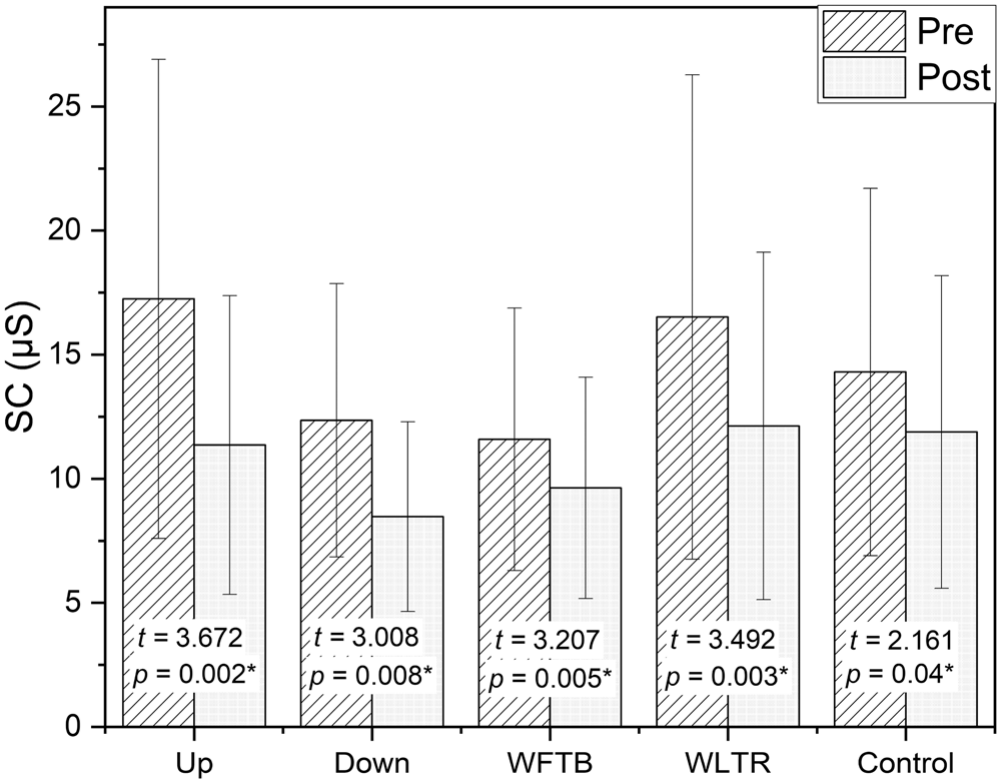
Pretest–posttest changes in skin conductance.

Considering Figure 3, it is apparent that a reduction in skin conductance occurred across all treatments with significantly different (*p* < 0.05). In this skin conductance variable, the largest mean difference was observed in the Up pattern, similar to the pulse rate decrease, followed by Down, WLTR, and lastly, the WFTB pattern. Detailed information regarding the changes in skin conductance as well as the corresponding effect sizes can be found in Table 4.

**Table 4:** Effect size of pretest–posttest changes in skin conductance.

	*N*	Pre mean (SD)	Post mean (SD)	Mean difference (95% CI)	*t*	*p*	*d*	Effect size
*SC*								
Up	19	17.25 (9.65)	11.36 (6.02)	5.89 (2.52, 9.25)	3.672	0.002*	0.66	Medium
Down	19	12.35 (5.51)	8.47 (3.82)	3.88 (1.17, 6.59)	3.008	0.008*	0.66	Medium
WFTB	19	11.59 (5.29)	9.63 (4.46)	1.96 (0.67, 3.24)	3.207	0.005*	0.36	Small
WLTR	19	16.52 (9.76)	12.12 (7.00)	4.40 (1.75, 7.04)	3.492	0.003*	0.47	Small
Control	19	14.30 (7.40)	11.89 (6.30)	2.42 (0.07, 4.77)	2.161	0.044*	0.32	Small

* *p* ≤ 0.05 (significant).

As shown in the figure, the error bars representing the standard deviation (SD) are very high; however, the pre–post changes consistently report significance. This occurs because the paired *t*‐test takes into account the difference between pre and post values for each individual rather than the total data variability. Even though the standard deviation for each group is large, if the difference between the pre‐ and post values is consistent for each individual, the paired *t*‐test results can still be significant. Additionally, a sufficiently large sample size can cause small but consistent differences across the sample to yield significant *p* values. Furthermore, in the presence of high data variability, substantial changes in certain individuals can dominate the analysis results, leading the *t*‐statistic to indicate significance. Thus, the paired *t*‐test focuses on evaluating differences within paired data rather than total group variation, explaining why pre–post changes remain significant despite a large SD.

### 3.2 Comfort Ratings

**Figure 4:**
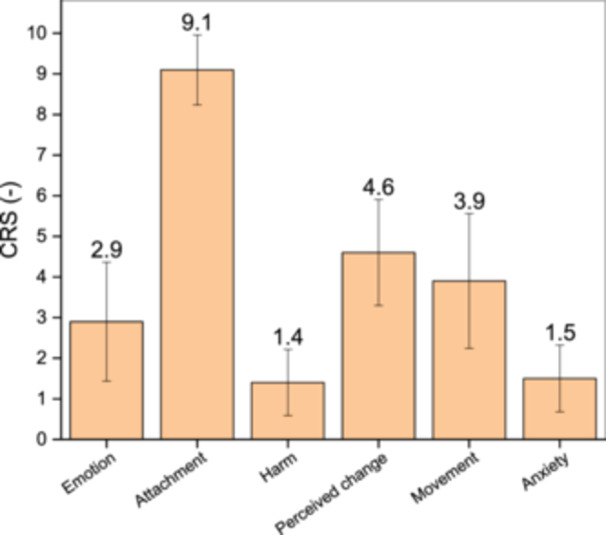
Comfort rating towards the vibrotactile‐weighted vest.

The comfort rating was conducted by 30 participants who had completed a series of studies, enabling them to subjectively assess the comfort provided by the vibrotactile‐weighted vest. Figure 4 indicates low ratings in the “Harm” category (1.4/10), “Anxiety” category (1.5/10), and “Emotion” category (2.9/10). Followed by “Movement” and “Perceived change” categories with medium ratings (3.9/10 and 4.6/10, respectively) then “Attachment” with a higher rating (9.1/10).

## 4 Discussion

In this study, vibrotactile‐weighted vest with different vibration directions were evaluated for the effectiveness in stimulating calmness by measuring decreases in pulse rate and skin conductance, and additionally assessed the safety and comfort of the device subjectively using CRS. The vibrotactile‐weighted vest is equipped with twenty‐coin vibration motors that apply four different types of vibration to the vest that can be controlled by the wearer. Vibrotactile stimulation was hypothesized to have a greater calming effect compared to weighted vest without vibration stimulation as indicated by a decrease in pulse rate and skin conductance.

This study indicates that the utilization of a vibrotactile‐weighted vest led to a significant reduction in participant's pulse rate and skin conductance across all groups. Specifically, a medium effect was observed in the Up and Down pattern, followed by a small effect in the WFTB and WLTR patterns. Even in the absence of vibrotactile stimulation, the group using the weighted vest exhibited a decrease in pulse rate and skin conductance, albeit to a lesser extent than the experimental groups. These results are in line with previous studies which reported that deep pressure therapy reduced physiological signs of stress or anxiety especially pulse rate and skin conductance [18]. Furthermore, we note that the vertical vibration pattern (Up and Down) consistently gives a medium effect both on variable pulse rate and skin conductance, then followed by horizontal pattern (WLTR and WFTB) with a small effect which indicates that the vertical patterns on the back provide a better calming effects followed the horizontal patterns on the waist, and this is in line with Morrison et al.'s report [19].

A previous study conducted by Azevedo et al. [29] has used wristband with heartbeat‐like vibration applied to enhance relaxation in participants who will be giving public speeches. Physiological measurements, including heart rate and skin conductance, were taken alongside subjective reports of anxiety. The study reported that vibrotactile stimulation significantly enhanced relaxation, as evidenced by reduced arousal in both physiological measures and participant's subjective anxiety reports. Those findings are in line with present studies which further strengthen the hypothesis that vibrotactile stimulation would reduce anxiety. Vibrotactile stimulation exerts a positive effect by triggering a hormonal response, specifically reducing cortisol levels, which are the end product of the sympathetic nerves active during stressful conditions [15, 24, 25, 30].

These findings are also consistent with recent research on psychophysiological recovery in high‐level managers, where training based on muscle relaxation and heart rate variability biofeedback led to significant improvements in various psychophysiological parameters [31]. This highlights the potential of multi‐modal approaches that combine physical and psychological interventions to target the complex nature of stress and anxiety.

The positive effect shown by physiological response is further strengthened by the low score domination on unfavorable statements (“Harm,” “Anxiety,” and “Emotion”), then medium score on “Perceived change” and “Movement.” Referring to Knight and Baber [20] we can conclude that the rating indicates that vibrotactile‐weighted vest is painless, not worrying, and safe when worn. The high score on “Attachment” indicates that the weight and vibration mounted on the vest are felt. It cannot be attributed to the negative response that Knight and Baber used, because the “attachment” in Knight and Baber's study interpreted that devices that feel attached and moving in the body are distractions, and must be reduced to get a good wearable device, whereas in deep‐pressure treatment studies, weight and vibration were applied to stimulate the parasympathetic nervous system which provides a calming effect [11, 24, 32, 33].

Medium score on “Perceived change” and “Movement” is an advance in the development of wearable devices as therapeutic tools, because in the previous form created by Biswas in the form of sleeping bags, users could only use it when sleeping so that it would automatically greatly inhibit user movement. But there needs to be more improvisation, especially on the slimmer control system so that the wearable device becomes very flexible and not flashy in appearance.

### 4.1 Limitations

The dyssynchronization of subjective comfort rating scores can be avoided by testing the validity and reliability of the questionnaire [20, 34] but this has not been done in this study. Another limitation is that no measurement of anxiety or stress scores has been carried out even though in the selection of respondents there has been an effort to homogenize the final group of semester students who are usually in a higher anxiety state than usual. Additionally, individual differences in body mass composition, which research suggests can influence physiological responses to stress and vibration therapy [35], were not accounted for in this study. Future research should include baseline assessments of stress and anxiety, as well as considering the potential moderating role of body mass composition on the effectiveness of vibrotactile stimulation.

### 4.2 Future Directions

Despite these limitations, the findings of this study have potential clinical implications. The vibrotactile‐weighted vest may offer a noninvasive, portable, and potentially effective tool for managing stress and anxiety in various settings, including clinical, educational, and workplace environments. Further research is needed to explore the long‐term effects and optimal parameters of vibrotactile stimulation, as well as to determine its efficacy in specific populations (e.g., individuals with anxiety disorders, autism spectrum disorder).

## 5 Conclusion

This study demonstrates the effectiveness of a vibrotactile weighted vest in reducing sympathetic activities as physiological signs of stress and rates as a safe and comfortable device. These findings suggest that vibrotactile stimulation may offer a promising noninvasive approach for managing stress and anxiety, warranting further investigation into its long‐term effects and optimal parameters.

## Author Contributions

**Mohamad Izzur Maula:** writing–original draft, investigation, funding acquisition, conceptualization, visualization, validation, methodology, writing–review and editing, project administration, formal analysis, software, resources, supervision, data curation. **Muhammad Imam Ammarullah** and **Farhan Ali Husaini:** resources, supervision, data curation, project administration, formal analysis, software, writing–review and editing, visualization, validation, methodology, writing–original draft, funding acquisition, investigation, conceptualization. **Chandra Maulana Nugwita:** writing–review and editing, visualization, validation, methodology, writing–original draft, funding acquisition, conceptualization, investigation, formal analysis, project administration, software, resources, supervision, data curation. **Muhammad Sultan Faisal:** resources, data curation, supervision, project administration, formal analysis, software, writing–review and editing, visualization, validation, methodology, funding acquisition, investigation, conceptualization, writing–original draft. **Ilham Yustar Afif:** funding acquisition, writing–original draft, investigation, conceptualization, methodology, validation, writing–review and editing, visualization, formal analysis, software, project administration, resources, data curation, supervision. **M. Danny Pratama Lamura:** funding acquisition, writing–original draft, investigation, conceptualization, methodology, validation, writing–review and editing, visualization, project administration, formal analysis, software, data curation, supervision, resources. **Jamari Jamari:** conceptualization, investigation, writing–original draft, funding acquisition, writing–review and editing, visualization, validation, methodology, software, formal analysis, project administration, resources, supervision, data curation. **Tri Indah Winarni:** writing–original draft, funding acquisition, investigation, conceptualization, methodology, visualization, validation, writing–review and editing, formal analysis, software, project administration, resources, supervision, data curation.

## Disclosure

The authors declare that this manuscript is original, has not been published before, and is not currently being considered for publication elsewhere. The authors confirm that the manuscript has been read and approved by all named authors and that there are no other persons who satisfied the criteria for authorship but are not listed. The authors further confirm that the order of authors listed in the manuscript has been approved by all of us. The authors understand that the corresponding author is the sole contact for the Editorial process. The corresponding author is responsible for communicating with the other authors about progress, submissions of revisions and final approval of proofs.

## Ethics Statement

This study involves human participants or animals, and ethical approval was required. All research procedures adhered to relevant ethical guidelines and best practices for nonhuman and nonanimal research. The study was conducted in accordance with the Declaration of Helsinki, and approved by the Health Research Ethics Committee of Diponegoro University (protocol number 373/EC/KEPK/FK‐UNDIP/IX/2021). This study is not a clinical trial and is therefore not registered in any trial registry.

## Consent

The participants provided written informed consent before participation in the study. They were informed about the study's purpose, procedures, potential risks, and their right to withdraw at any time without penalty. The study was conducted in accordance with the Declaration of Helsinki and was approved by the Institutional Review Board (IRB) of Universitas Diponegoro.

## Conflicts of Interest

The authors declare no conflicts of interest.

## Transparency Statement

The authors affirm that this manuscript is an honest, accurate, and transparent account of the study being reported; that no important aspects of the study have been omitted; and that any discrepancies from the study as planned (and, if relevant, registered) have been explained.

## Declaration of AI use

The authors declare that they did not use AI‐assisted technologies in creating this article.

## Data Availability

The necessary data used in the manuscript are already present in the manuscript.
